# Comparison of Potato Viromes Between Introduced and Indigenous Varieties

**DOI:** 10.3389/fmicb.2022.809780

**Published:** 2022-05-04

**Authors:** Xianjun Lai, Haiyan Wang, Caiyun Wu, Wen Zheng, Jing Leng, Yizheng Zhang, Lang Yan

**Affiliations:** ^1^Panxi Crops Research and Utilization Key Laboratory of Sichuan Province, College of Agricultural Science, Xichang University, Xichang, China; ^2^Sichuan Key Laboratory of Molecular Biology and Biotechnology, College of Life Sciences, Sichuan University, Chengdu, China

**Keywords:** potato, virome, RNA-seq, viral genome, mutation

## Abstract

Viral disease in potatoes has been a major problem in potato production worldwide. In addition to the potential risk of introducing new diseases in new areas, viral-disease epidemics/pandemics can be initiated by “spillover” of indigenous viruses from infected alternative hosts into introduced cultivars. To investigate the tendency of potential viral infection/resistance, we analyzed the viromes of introduced and indigenous varieties of potatoes among different tissues using RNA-seq libraries. Bioinformatics analyses revealed that potato viruses PVM, PVY, and PVS were dominant and the most frequently identified viruses infecting potato virus-free plants in the field, and showed an infection bias between introduced and indigenous cultivars. PVY and PVS were the major viruses in introduced varieties, whereas PVM showed an extraordinarily high percentage in the indigenous variety. Other three common viruses, PVH, potato mop-top virus, and potato leafroll virus were identified specifically in the indigenous variety. There was a tendency for tissue-specific infection and sequence variation in viruses: underground parts (tubers, roots) harbored more unusual viruses, and tubers harbored relatively more variation with a high frequency of single nucleotide polymorphisms than other tissues. Taken together, our study provides a comprehensive overview of the composition, distribution, and sequence variation of viruses between introduced and indigenous varieties of potatoes.

## Introduction

More than 100 countries cultivate potatoes. It is an essential staple food and vegetable crop and ranked fourth in production after rice, wheat, and maize (Bond, 2014). China holds the first position in the potato-production industry, with ∼90 million tons of potatoes being produced from 4 million ha of land in 2018 (According to the FAO). Optimization of production levels and resistance to biotic and abiotic stresses are the fundamental objectives of potato-breeding programs. However, viruses are major constraints on potato production systems (Devaux et al., 2020; Kreuze et al., 2020), and can cause losses in tuber yields ≤50% (Wale et al., 2008). Due to the vegetative propagation that supports viral transmission over successive generations, potatoes are susceptible to many damaging viral diseases (Locke, 2002; Jones, 2014; Kreuze et al., 2020). More than 40 potato viruses have been reported worldwide, with 16 reported in China alone (Jeffries et al., 2005; Rashid et al., 2021). The potato viruses X (PVX), Y (PVY), S (PVS), A (PVA), M (PVM), and potato leaf roll virus (PLRV) are the main pathogens responsible for most yield reductions (Zhang et al., 2010; Awasthi and Verma, 2017; Kreuze et al., 2020).

Special interest has arisen from the study of potato-virus interactions that occur with landraces or introduced species worldwide. Potatoes were first brought from Andean crop domestication center to Europe in the 16th century, after which the crop was taken to other continents (Glenndinning, 1983; Nunn and Qian, 2010). Unknowingly, potato virus was also introduced to new locations through trade in infected potato tubers and continues to evolve with the relatively recent emergence of new damaging recombinant strains (Torrance and Talianksy, 2020). Development of damaging virus epidemics is favored by the introduction of new germplasm to parts of the world where they have never been grown before, which lead to new encounters with virulent viruses infecting indigenous cultivars or land races (Thresh, 1982; Loebenstein and Thottappilly, 2013). When viruses coevolve for a very long period locally within potato native landrace, a high degree of nucleotide sequence diversity occurs amongst isolates collected over a certain small geographic range, which comes into the threaten to subsequent introduced potatoes or non-potato hosts (Spetz et al., 2003; Jones, 2009). As reported, several of the world’s plant virus disease epidemics arose from viruses spread by spillover (host species jumps) from infected indigenous plants to infect introduced cultivated plants, rather than virus spread from introduced crops to indigenous crops or natural vegetation (Roossinck and García-Arenal, 2015). The term “virus spillover” refers to spread of indigenous virus variants associated with naturally-infected host to the introduced host it has not encountered previously (Jones, 2020). The outcome of virus spillover for each individual variant depends on its relative abilities (i.e., fitness) to survive once it infects each host, adapt to new hosts or vectors and achieve efficient epidemic spread (Harlan, 1971).

Studying the exchange of viruses between native landraces and introduced cultivars is a neglected area that has the potential to provide information on threats to introduction cultivation and biodiversity, as well as virus mutation and evolution. Liangshan Yi autonomous prefecture, a remote, impoverished, and mountainous area in southern China, has been planting potatoes for more than 100 years and nurtured many unique potato landraces because of its unique natural environment (Li et al., 2013). What exist there today are diverse potato community with scattered patches of an endemic, mixed with the recently introduced potato germplasm of low biodiversity sourced from northern China or abroad (Peng and Luo, 2010). This interface between indigenous and introduced potato communities is ideal for studying interactions between virus variants of an isolated indigenous flora and introduced cultivars. Given the fact that virus is capable of extending its natural host range under field conditions (Luo et al., 2011), more attention should be paid to studying whether the indigenous potato virus has selective invasion to the introduced cultivars and how they mutate and coevolve amongst isolates.

In recent years, various detection approaches have been established for identification of plant viruses (including potato viruses) (Xu et al., 2017; Cao et al., 2019; Rashid et al., 2021). In addition to symptomatology and serological methods, molecular diagnostic tools facilitate virus identification (Maclot et al., 2020). Several next-generation sequencing (NGS)-based approaches, including RNA-sequencing (RNA-seq) and small RNA-sequencing (sRNA-seq) have been used widely for studies on plant viromes (Bejerman et al., 2020). The most commonly utilized approach is total sRNA-sequencing (total RNA-seq) because the double-stranded-RNA in plant cells is processed into small interfering RNA (siRNA) molecules to target viral RNA for degradation during viral infection, sRNA preparations can be analyzed for viruses populating the host (Roossinck et al., 2015). However, viruses that do not induce RNA-silencing mechanisms or counteract the plant’s defense mechanism by activating suppressor genes might not be identified by sRNA-sequencing. Total RNA-seq may be required for characterization of the virome, which also enables the assembly of individual viral genomes and reveals the nucleotide variations of viruses (Pecman et al., 2017).

Here we identified potato viromes using total RNA-seq. Twenty-two libraries were prepared from the leaves, stems, roots, stolons, and tubers collected from five cultivars in the native landrace of Liangshan and introduced varieties from Russia, of which potato plants from virus-free tissue-culture breeding systems re-infected and co-infected by indigenous viruses after transplantation to the field. We wished to address the impact of indigenous viruses on native and introduced potato germplasm through comparing the viromes of different varieties among different tissues, and characterize the host-specific viral communities in a tissue-level atlas. Comprehensive bioinformatics analyses revealed the complexity and unique viral communities associated with various factors, which will lay the foundations of virus disease-control strategies for future production of introduced cultivated potatoes.

## Materials and Methods

### Collection of Potato Samples, RNA Extraction, and Transcriptome Sequencing

We collected totally five potato varieties: four introduced cultivars from Russia named “Rosa,” “Nevsky,” “Talovskij,” and “Pioneer,” respectively, and one native landrace “S24” from Liangshan Yi autonomous prefecture, the southwest mountainous area in China. The germplasms introduced from Russia were obtained from the China National Potato Improvement Center (Keshan, Heilongjiang, China). They passed rigorous quarantine and were in the form of virus-free tissue-cultured small plants. Similarly, virus-free plants of “S24” were obtained by culture of shoot tips. The original potatoes grown from these virus-free plantlets in the substrate pot were planted in the field, and the recovery of plants from virus infection among different varieties had been detected. In the harvesting period, we collected five tissues involving in leaves, stems, stolons, roots, tubers from each cultivar. Unexpectedly, one of the cultivars, Pioneer, did not develop tubers, and the stolons and roots of Talovskij were rotted upon sampling. As a result, 22 samples were used for RNA extraction.

Fresh tissues from each sample were ground in Hanks’ buffered salt solution (1:10) with four ceramic beads using a tissue homogenizer (MP Biomedicals, Solon, OH, United States). Then, total RNA was extracted using the RNeasy Plant Mini Kit (Qiagen, Hilden, Germany) in accordance with manufacturer instructions. The quantity of extracted RNAs was detected using a spectrophotometer (NanoDrop, Thermo Scientific, Waltham, MA, United States) and the quality was measured by gel electrophoresis. For library construction of RNA sequences, the NEBNext Ultra RNA Library Prep Kit for an Illumina platform was used according to manufacturer instructions (New England Biolabs, Ipswich, MA, United States). Transcriptome sequencing was done on a HiSeq 2500 system (Illumina).

### *De novo* Transcriptome Assembly and Identification of Virus and Viroids

Bioinformatics analyses involved transcriptome assembly, reads mapping, and BLAST searching. A workstation with two eight-core CPUs and 192 GB of RAM installed with a Linux Mint 19.2 system was used for these tasks. Adaptors and low-quality reads were filtered first using Trimmomatic^1^ (Bolger et al., 2014) and two strategies were applied: align-then-assemble and assemble-then-align. The align-then-assemble strategy was implemented mainly by Bowtie 2.0.2^2^ (Langmead and Salzberg, 2012) and TopHat 2.0.6^3^ (Kim et al., 2013). Unmapped reads from each individual were assembled by Trinity 2.0.2 (Grabherr et al., 2011). In the assemble-then-align strategy, all cleaned RNA-seq reads were assembled *de novo* by Trinity using the same parameters.

To identify virus- and viroid-associated contigs, the assembled contigs from different strategies were combined against the viral reference database of the National Center for Biotechnology Information^4^ and viroid database^5^ using MEGABLAST with a cutoff E-value of 1 × 10^–5^ (Morgulis et al., 2008; Lee et al., 2022). After filtering the phage, endogenous virus-like sequences, and other contaminated sequences through manual checking, the obtained virus-associated contigs were aligned to the genome of the reference virus. The results were visualized through Tablet software^6^ (Milne et al., 2013).

### Phylogenetic Analyses of Identified Viruses

For analyses of phylogenetic trees, the genome sequences of viruses assembled in our study and the available viral-genome sequences from GenBank were used. Based on BLAST results, the virus-associated contigs in each library were aligned on the identified genomes of the virus reference using ClustalW^7^ with default parameters (Larkin et al., 2007). Twenty-nine PVM genomes, 40 PVY genomes, 33 PVS genomes, 17 PLRV genomes, 31 PMTV genomes, and 15 PVH genomes for which we had nearly full-length genome sequences were used. Instead of conserved RNA-dependent RNA polymerase (RdRp) or coat protein (CP) amino sequences, we used complete genome sequences and their homologous sequences for construction of phylogenetic trees. We aligned the genome sequences for each group of viruses using MAFFT^8^ with default parameters (Katoh and Standley, 2013) and eliminated all positions containing gaps or missing data. Sequences were tested for recombinants using the full suite of options (six available recombination analysis programs including RDP, GENECONV, Chimaera, MaxChi, Bootscan, and SiScan) in Recombination Detection Program (RDP, version 5.05) with default parameters (Martin and Rybicki, 2000; Martin et al., 2021). Anomalies found by five or fewer methods and with greater than 10^–5^ random probability were ignored.

The phylogenetic tree was constructed via the statistical method of maximum likelihood (ML) using phyML 3.0^9^ (Guindon et al., 2010), with parameters estimated from the data. The general time reversible (GTR) substitution model was selected for ML analyses taking into account the gamma distribution of rate heterogeneity with four discrete categories. Branch support was evaluated by 1,000 replications of bootstrap re-sampling (Yang, 1994). The generated phylogenetic trees for individual viral species were visualized using FigTree 1.4.4.^10^

### Analyses of Single-Nucleotide Polymorphism Variation for Each Viral Genome

To identify the mutation positions of identified viruses, we detected SNPs for the identified viral genomes among different cultivars and tissues. The assembled genome sequences of six virus PVS, PVY, PVM, PMTV, PLRV, and PVH were used as reference virus genome sequences. We carried out SNP analysis as described previously (Jo et al., 2018). In brief, we aligned the raw sequence reads in each library on the assembled viral genome using the BWA program with default parameters (Li and Durbin, 2009), which resulted in SAM files. We converted the SAM files generated by BWA into BAM files via SAMtools (Li et al., 2009). Next, the sorted BAM files were converted into the variant call format (VCF) file format using the mpileup function of SAMtools for SNP calling. Finally, BCFtools was implemented in SAMtools to call SNPs. The positions of identified SNPs on each viral genome were visualized by Tablet (Milne et al., 2013).

### Calculation of the Virus Accumulation and Copy Number

To quantify the virus-related RNAs associated with identified viruses in each library, we calculated the number of sequenced reads associated with respective viruses contigs via MEGABLAST search (Morgulis et al., 2008). At the same time, the raw reads were mapped onto the identified viral contigs using Bowtie2 (Langmead and Salzberg, 2012) to estimate viral contigs abundance, which called alignment-based abundance estimation methods used for RNA-Seq (Li and Dewey, 2011). To calculate the copy number for each virus, the obtained number of virus-associated reads was multiplied by 150 and then divided by the total length of the individual genome (Jo et al., 2018).

### Confirmation of the Viruses Comprising the Viromes

For the confirmation of the viruses obtained by the NGS analysis and for the future application of the virome results, we conducted RT-PCR for three viruses PVS, PVY and PVM with specific primers (Supplementary Table 4). Total RNA was treated with DNase I (Takara Bio, Shiga, Japan) and reverse-transcribed using the PrimeScript Reverse Transcription reagent Kit (Takara Bio, Shiga, Japan) following the manufacturer’s instructions. PCR reaction were performed in a Bio-Rad T100™ Thermal Cycler (Bio-Rad Laboratories, Hercules, CA, United States) using PrimeScript RT-PCR Kit (Takara Bio, Shiga, Japan). Amplified fragments were sequenced in Sangon Biotech (Shanghai) Co., Ltd. to confirm the viral sequence.

### Data Availability

The raw data of RNA-seq in this study have been deposited in the NCBI database under BioProject number PRJNA760655.

## Results

### Collection of Cultivar and Tissues for Identification of Potato Viromes

We wished to identify viral populations inhabiting potato varieties from different germplasm sources and the established viromes of multiple tissues. Introduced and indigenous potato plants were grown in the same field. Potatoes showing disease symptoms were sampled. Finally, we collected the leaves, stems, roots, stolons, and tubers from four cultivars introduced from Russia (“Rosa,” “Nevsky,” “Talovskij,” “Pioneer”) and one native landrace (“S24”) from Liangshan. Samples were pooled based on the varieties and tissues. Twenty-two libraries for RNA-seq were prepared and paired-end sequenced by the HiSeq150 system (HiSeq, San Diego, CA, United States). Three libraries (tubers of Pioneer, stolons and roots of Talovskij) were absent due to a failure of tissue sampling. The raw sequence data for RNA-seq are listed in Supplementary Table 1.

### Transcriptome Assembly and Virus Identification

**FIGURE 1:**
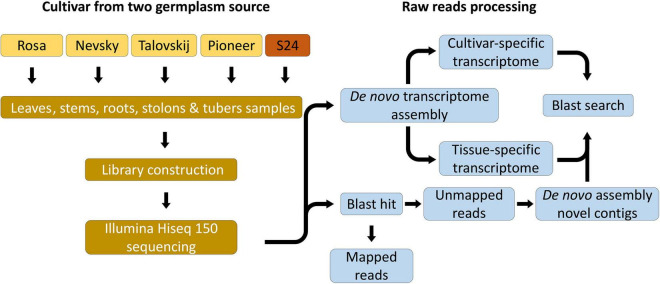
Scheme of bioinformatic analyses for the 22 potato viromes. Two strategies, “align-then-assemble” and “assemble-then-align,” are described.

To identify the virus-related sequences as accurately as possible, two strategies were combined: “align-then-assemble” and “assemble-then-align” (Figure 1). Based on the assemble-then-align strategy, RNA-seq reads from each sample were individually assembled *de novo* using Trinity^11^ with the assembled contigs (transcripts) ranging from 98,039 to 144,588 and contig N50 ranging from 1,584 to 1,862 (Supplementary Table 2). Then, the individual transcriptomes were combined according to the cultivar and tissue, respectively, to form five cultivar-specific and other five tissue-specific transcriptomes. Finally, the contigs obtained from these transcriptomes were aligned against the genome sequences of the plant-virus reference derived from a viral genome database (see text footnote 4) and viroid database (see text footnote 5) through MEGABLAST search. The align-then-assemble strategy involved the mapping to the genome of the potato reference. Results showed that 27.8% reads failed to the BLAST hit. Then, these unmapped reads were assembled *de novo* into novel contigs through Trinity. Finally, an average of 84,119 contigs with contig N50 of 775 bp and a total length of 51.51 Mb were obtained.

The results of BLAST searching from above two strategies were combined, total of which 11 representative potato viruses and one viroid involving 1,047 virus-related contigs were identified (Supplementary Table 3). To confirm the RNA-Seq results and to develop molecular diagnostic methods for viruses infecting potatoes, we examined infection of virus in 22 samples by RT-PCR and using virus-specific primer pairs for three viruses: PVY, PVM, and PVS (Supplementary Table 4). Amplified PCR products from each sample were visualized by gel electrophoresis (Supplementary Figure 1). In general, RT-PCR results were correlated with those of RNA-Seq, in which PVY was identified from all 22 libraries and PVM was identified in all cultivars except “Pioneer”.

### Distribution of Viral Infection Among Cultivars and Tissues

**FIGURE 2:**
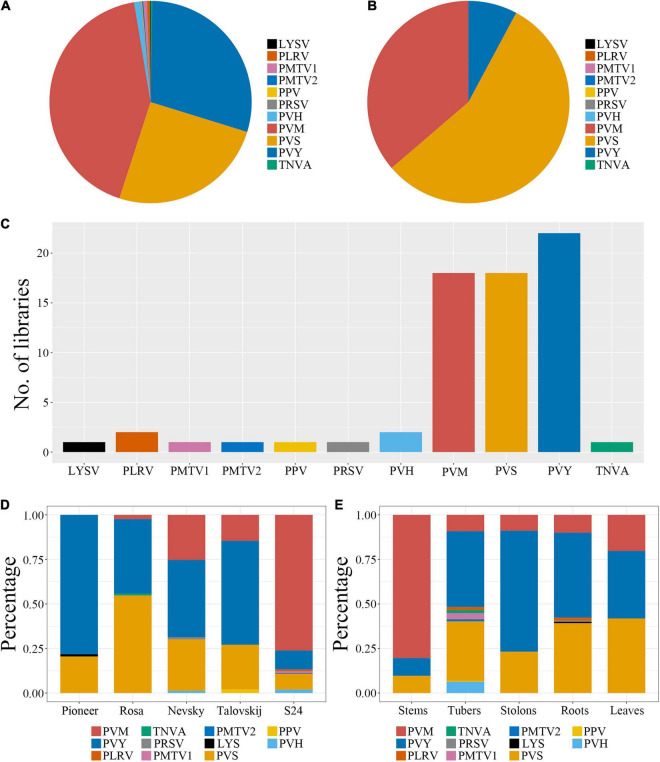
Identification of viruses infecting potatoes and proportion of identified viruses in each library. **(A,B)** Proportions of identified viruses from all 22 libraries based on virus-associated contigs and reads, respectively. **(C)** Number of libraries for an individual identified virus. **(D,E)** Proportion of identified viruses among different cultivars and tissues, respectively, based on virus-associated contigs.

First, we analyzed the proportion of identified viruses from all libraries. Based on virus-associated contigs, PVM (437 contigs) was the dominant virus, followed by PVY (304 contigs) and PVS (206 contigs) (Figure 2A). In consideration of the total virus-associated reads, PVS (52 million reads) was the major virus infecting sweet potato, followed by PVM (34 million reads), and PVY (7 million reads) (Figure 2B).

Next, we examined the number of samples for each individual identified virus. Among 22 libraries, the most frequently identified virus was PVY (which resided in all libraries), followed by PVM and PVS (both of which were present in 18 libraries). According to virus-associated contigs, the virus composition was essentially identical among different cultivars, and PVY, PVS, and PVM were dominant. However, the proportion of viruses showed significant differences between introduced and indigenous varieties (Figure 2C). PVY was the major virus in four introduced varieties, and occupied 78.2% in Pioneer and 41.8–58.3% in the other three varieties, but only 10.6% in S24. PVS also showed a relatively high proportion in introduced varieties, was dominant in Rosa (54.7%), abundant in the other varieties (>20%), but only 8.9% in S24. PVM showed an extraordinarily high percentage (76.1%) in S24 but only 14.5–24% in the introduced varieties, and was not identified in Pioneer (Figure 2D). These results demonstrated that, although the main types of viruses infecting different potatoes were similar, the degree of infection between introduced and indigenous varieties caused by these viruses having a dominant role were different. Some virus-associated contigs, such as potato virus H (PVH; 10 contigs), potato mop-top virus RNA (PMTV; 8 contigs) and potato leafroll virus (PLRV; 5 contigs) were identified specifically in S24. In addition, one viroid named citrus exocortis viroid was identified in S24 with 5 viroid-associated contigs and introduced varieties “Rosa” (3 contigs), “Nevsky” (4 contigs) and “Talovskij” (4 contigs).

With regard to virus distribution among tissues, the three dominant viruses (PVY, PVS, and PVM) comprised the whole viromes of leaves, stems, and stolons. PVM was the dominant virus in stems (80.4%) and PVY was the major virus in stolons (67.85%). The proportions of the three viruses in leaves were distributed relatively evenly, with PVS reaching 41.85%. In addition to PVY, PVS, and PVM, some unusual viruses, such as PLRV, papaya ringspot virus (PRSV) and leek yellow stripe virus (LYSV), were identified in roots. As expected, tubers harbored more unusual viruses, in which PLRV, tobacco necrosis virus A (TNVA), PMTV, and PVH were identified, although the levels were very low (Figure 2E).

### Phylogenetic Relationships of the Identified Viruses Based on Complete Genomes

**FIGURE 3:**
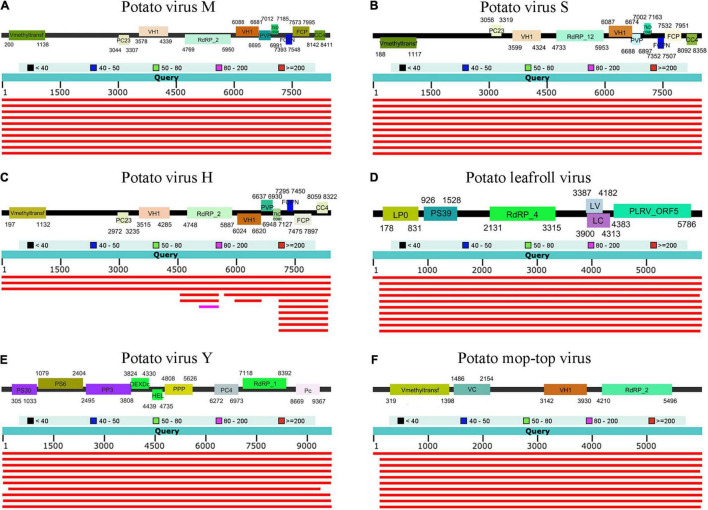
Genome organization of **(A)** PVM **(B)** PVS **(C)** PVH **(D)** PLRV **(E)** PVY and **(F)** PMTV with virus-associated contigs covered nearly the complete genome. BLASTN results against the NCBI nt database are described.

To assemble the complete (or nearly-complete) genomes of identified viruses, virus-associated contigs were mapped on each reference viral genome. Contigs associated with PVM, PVY, PLRV, PMTV, PVS, and PVH covered nearly the complete genomes, whereas TNVA, PRSV, LYSV, and XMRV-1 were partially mapped by the virus-associated contigs (Figure 3).

**FIGURE 4:**
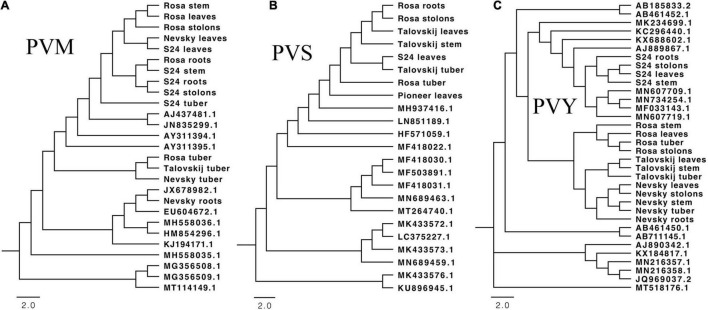
Maximum-likelihood phylogenetic tree of genome sequences for three main identified virus isolates: PVM **(A)**, PVS **(B)**, and PVY **(C)**. The accession number of known isolates is described. We used bootstrap replication values of 1,000. Bootstrap values > 70% are shown.

Representative viral sequences of PVS, PVM, and PVY were then examined for phylogenetic anomalies using the full suite of options in RDP. Nine PVS sequences gave significant evidence of recombination. PVM had rarely recombinants and only one recombinant sequence was found. PVY exists as a complex of strains, including a growing number of recombinants. We identified twelve PVY sequences in RDP analyses which were recorded as significant recombinants (Supplementary Figure 2). After removing the recombination sequences in PVS, PVM, and PVY, we constructed phylogenetic trees to reveal the phylogenetic relationships between these identified viruses and other known isolates. Only complete or nearly complete assembled viral genome sequences were matched to known viruses for phylogenetic analyses. PVM showed the tissue-specific variation, in which the tuber-PVMs from introduced varieties clustered together and had a relatively distant relationship with that from S24. In addition, PVM in S24 and other tissues of introduced varieties were closely related to known isolates from Hangzhou, China (AJ437481.1) and Gansu, China (JN835299.1), in comparison of other known isolates (Figure 4A). Due to that the recombination sequences were removed, PVS had relatively fewer identified viruses in the phylogenetic tree. In spite of that, isolate from S24 was close related to the introduced varieties (Figure 4B). PVY has been found to have several mutant strains in previous studies (Della Bartola et al., 2020) and we also found strains different from known isolates (Figure 4C). Unlike PVM showing tissue-specific variation, PVY in multiple tissues of the same variety were grouped together. PVY from S24 had close relationship with the known isolates from multiple areas in China and were separated clearly from the other three introduced varieties. In addition, phylogenetic trees also showed that PLRV (which was found specifically in S24) was highly homologous and closely related to the PLRV-IM isolate from Inner Mongolia. The native-specific viruses PMTV (PMTV1 and PMTV2) showed strong sequence similarity with segments RNA1 and RNA3 from the Yunnan isolate, and PVH was closely related to the Yunnan isolate, too (data not shown).

### Mutation Rates and Single-Nucleotide Polymorphism Variations for Individual Viruses

To determine virus variation among different cultivars and tissues, we mapped raw sequence reads on the six viral genomes in which nearly complete genomes were assembled from our study. Simultaneously, raw sequence reads from each transcriptome were aligned to the assembled viral genomes for Single-Nucleotide Polymorphism (SNP) calling.

**FIGURE 5:**
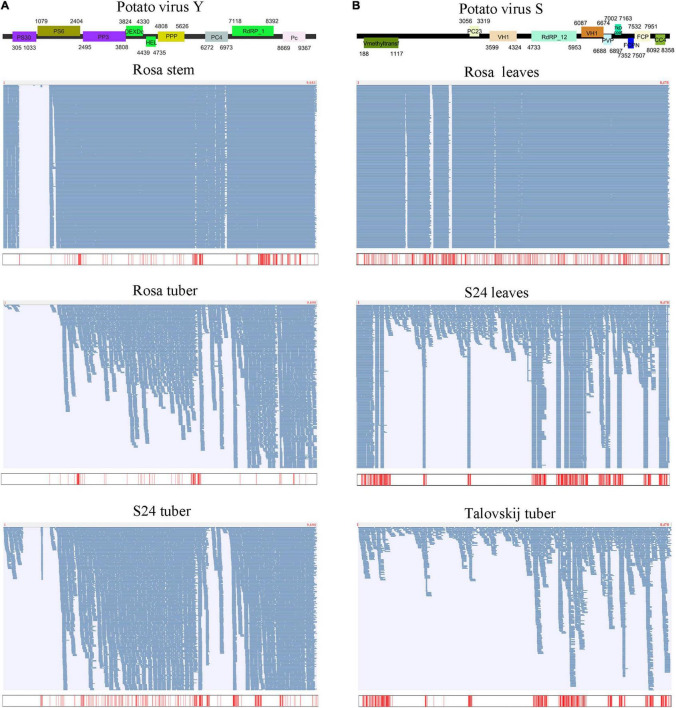
Assembly of viral genomes and SNP analyses for PVY **(A)** and PVS **(B)**. The upper part shows the organization of the assembled viral genome. The lower part shows the mapping results of sequence reads on the assembled viral genome among different cultivars and tissues, and the positions of identified SNPs are shown below.

First, we examined the mapping patterns of sequence reads on an individual virus. The whole regions of most viral genomes were mapped by reads without gaps. Interestingly, for most viruses identified from tubers, the number of mapped reads increased from the 5’ region to the 3’ region of the viral genome (Figure 5). Next, we examined the percentage of SNPs in each viral genome. Although SNPs were identified in six types of virues (PVS, PVY, PVM, PMTV, PLRV, and PVH), only PVS and PVY showed relatively high percentages of SNPs, ranging from 0.01 to 5.07%. Eleven PVS isolates showed many SNPs, of which 430 SNPs were identified from the leaves of S24, followed by 390 from the tubers of Nevsky, 365 from the leaves of Rosa and tubers of Talovskij. PVY also showed a high percentage of SNPs in Rosa, ranging from 0.89% (tubers) to 2.24% (roots). Although PVY isolated from all tissues of S24 also showed SNPs, only viruses in tubers showed a high level of 2.58%, whereas virus in other tissues showed a low percentage of SNPs, ranging from 0.02 to 0.05%.

### Virus Accumulation and Co-infection Among Potato Cultivars

To identify co-infections of viruses among different cultivars and tissues, we built a matrix to calculate the correlation coefficient between different viruses through normalizing the number of reads related to different viruses in each library because the number of sequenced reads associated with identified viruses in each transcriptome differed. Interestingly, the leaves, stems, and tubers of Rosa and S24 accumulated many virus-associated reads (31.08–69.78%), whereas the proportion of other cultivars and tissues was relatively low (0.002–8.28%).

In addition, we calculated the copy number (also known as the “sequence coverage”) of each identified virus based on the number of virus-associated reads. The results for the copy number were similar to those for virus accumulation, and the infective patterns of different viruses were identified readily. PVM was enriched specifically in S24, whereas virus accumulation decreased by three orders of magnitude in the introduced varieties. PVM in S24 was co-infected among different tissues, of which the copy numbers ranged from 93,389 in roots to 253,408 in leaves. Cultivar-specific infection was more prominent for PVS because only Rosa had extremely high virus accumulation among all three introduced varieties, the copy number of which ranged from 179,764 in roots to 230,648 in leaves. Nevertheless, PVS co-infected S24 relatively mildly, with the copy numbers ranging from 643 in roots to 5,349 in leaves. However, PVY infection seemed to be tissue-specific instead of cultivar-specific because the virus co-infected almost all varieties but had only a high degree of accumulation in leaves and stems, therefore it did not infect a certain variety in a concentrated manner.

## Discussion

Virome research has gradually become a hot spot in virus research and has been reported in a number of crops and fruits (Jo et al., 2017, 2018, 2020; Pecman et al., 2017). Although these studies based on NGS have identified known or novel viruses, comprehensive studies associated with potato viromes have been scarce. In this study, we carried out comprehensive analyses on potato viromes involving multiple tissues of introduced and indigenous cultivars using transcriptome analyses, identifying the dominant viruses infecting potato virus-free plants in the field, which showed a bias in infection between introduced and indigenous cultivars. Originally, we planned to conduct a multi-sample population analysis of introduced and indigenous varieties on potato viromes. However, compared with the introduced varieties, the indigenous varieties had better environmental adaptability and resistance to viruses: only one susceptible variety showed virus-infected symptoms. Nevertheless, a particular pattern in the composition, distribution, and sequence variation between introduced and indigenous varieties was documented. In this way, our study can be a reference for resistance-and-adaptation exercises of introduced cultivars in the future. Here, we addressed some key points associated with potato viromes and viral communities that differ from those described previously.

In general, studies have focused on the effects of introduced and indigenous viruses on native plants because importation of infected plants in a new area is a risk for disease emergence, and because newly introduced viruses can invade communities of native plants (Davis and Guy, 2001; Alexander et al., 2014). In potatoes, *in vitro* culture methods have been applied for production of virus-free plants (Wang et al., 2018). It is necessary to control viral diseases if importing novel cultivars from other countries and to exchange breeding materials between countries or regions. However, compared with virus spread from introduced cultivars to indigenous cultivars or natural vegetation, the risk of epidemics of viral disease arising from new encounters between indigenous viruses and introduced cultivars planted in the field seems higher than expected. For instance, the most economically important viral disease in tomatoes is tomato yellow leaf curl disease (TYLCD), attributed from tomato yellow leaf virus (TYLCV) infection (Mabvakure et al., 2016). TYLCV is a virus indigenous to the Middle East, and the tomato was domesticated in the Andean Region of South America. However, TYLCD emerged in a new-encounter scenario somewhere between the Jordan Valley and eastward to Iran. This emergence occurred by spillover from unidentified indigenous TYLCV-infected host sources into the introduced tomato crop (Lefeuvre et al., 2010). To make matters worse, TYLCV had generated virulent new variants through mutation, reassortment, and recombination in the process of spreading (Monci et al., 2002). In this study, we revealed the list of coinfected viruses in multiple tissues of introduced and indigenous potatoes, presenting particular patterns in the virus composition, distribution, and sequence variation and recombination. As identified in our study by phylogenetic-tree analyses, some viruses mutated between the indigenous and introduced cultivars, indicating that spillover starts with efficient epidemic spread of already existing virus variants from a virus-infection source to a new host and is dependent upon the relative ability and adaptation to new hosts of an individual variant. Moreover, selection of the proper tissue enriched with viruses is necessary for a successful virome study. Previous studies demonstrated that viral RNA was enriched in tuberous roots of sweet potato and grape fruits (Jo et al., 2015, 2020). Of course, the optimal tissues for plant virome studies depend on the plant species. In our study, the dominant viruses were distributed mainly in stems, stolons, and leaves, which indicating that tuber tissues might not be appropriate samples for the detection of viruses infecting potatoes. Also, a tendency for tissue-specific infection and sequence variations in viruses was identified that tubers harbored relatively greater variation with a high percentage of SNPs than that in other tissues.

Recombination, reassortment, and accumulation of mutations are the main forces shaping the evolution of viruses, with recombination being one of the main factors proceeding more rapidly. From the results in this study, the viruses infected in the introduced varieties undergo extensive recombination, which indicating that viral recombination was not relatively rare events. In other words, recombination between different strains of viruses is relatively frequent. Recombinant strains of PVY are prominent among three main viruses in this study, followed by PVS. After reconstructing the pathway of emergence of PVS and PVY recombinants, we found that the recombination sources of viruses in the introduced varieties are relatively conservative, basically from mutual recombination and sequence fragments derived from native landrace S24 (Supplementary Figure 2). Therefore, the role played by recombination and pseudo-recombination in potato viruses adapted to introduced cultivars as a new host was well established in this study.

Potato is widely grown in China, from south (Hainan) to north (Inner Mongolia) and from east (Zhejiang) to west (Tibet). Potato viral diseases, particularly PLRV, PVA, PVM, PVS, PVX, and PYV, are a limiting factor for sustainable production of potato in China because of the significant tuber yield losses they cause. Due to potatoes have been spread to Liangshan for more than 100 years, and there is not much communication with the outside due to traffic congestion, the evolution and mutation of local viruses are worthy of in-depth study. From the results of this study, the native landrece S24 was mainly infected by PVM, PVS, and PVY, and showed significant abundance differences among tissues. As reported by former researchers, the detection rates of PVX and PVA viruses were relatively high, reaching 17.74 and 16.41%, while the detection rates of PVY were below 10% in the main potato producing area of Sichuan Province, southwest China (Zhong et al., 2008). However, in Liangshan, southwest of Sichuan Province, neither PVX nor PVA virus was detected, while PVY was considered to be the main virus infecting local and exotic species. This suggests that the Liangshan ecoregion is a unique research environment for potato virus infection, mainly due to its high altitude (average elevation in Liangshan is 1,800 m, while the rest of Sichuan province has an average elevation of 500 m). In future research, the traceability and evolution of local viruses in Liangshan should be further explored, including the widescale distribution of infected planting material, recombination generating virulent new variants, synergistic interactions resulting from mixed infections, and how these viruses reach super-abundant numbers even above 1,000 m above sea level.

## Data Availability Statement

The datasets presented in this study can be found in online repositories. The names of the repository/repositories and accession number(s) can be found in the article/Supplementary Material.

## Author Contributions

LY, YZ, and XL designed the research. XL, CW, and WZ prepared the plant sample. XL and HW performed the transcriptome sequencing, assembly, and annotation. XL and JL performed the virus identification and variation analysis. YZ and HW supervised the experiments and analysis and revised the manuscript. LY and XL wrote the initial draft of the manuscript. All authors read and approved the final manuscript.

## Conflict of Interest

The authors declare that the research was conducted in the absence of any commercial or financial relationships that could be construed as a potential conflict of interest.

## Publisher’s Note

All claims expressed in this article are solely those of the authors and do not necessarily represent those of their affiliated organizations, or those of the publisher, the editors and the reviewers. Any product that may be evaluated in this article, or claim that may be made by its manufacturer, is not guaranteed or endorsed by the publisher.

## Footnotes

1
www.usadellab.org/

2
http://bowtie-bio.sourceforge.net/

3
http://ccb.jhu.edu/software/tophat/index.shtml

4
www.ncbi.nlm.nih.gov/genome/viruses/

5
https://viroids.org/

6
https://ics.hutton.ac.uk/tablet/

7
www.ebi.ac.uk/Tools/msa/clustalo/

8
https://mafft.cbrc.jp/alignment/software/

9
http://www.atgc-montpellier.fr/phyml/

10
https://github.com/rambaut/figtree/releases/

11
https://github.com/trinityrnaseq/

## Supplementary Material

Supplementary Figure 1 Confirmation of 3 identified viruses infecting potato by RT-PCR. Agarose gel electrophoresis results of **(A)** PVY, **(B)** PVM, and **(C)** PVS by RT-PCR with virus-specific primer pairs. Noted: the tubers of “Pioneer”, stolons and roots of “Talovskij” were failed in sampling and further RNA extraction and RT-PCR.

Supplementary Figure 2 A schematic diagram of the recombinant structures in the combined **(A)** PVM, **(B)** PVY, and **(C)** PVS datasets. Regions of recombination are marked by colored bar, and fragments originating from different parents are colored accordingly.
