# Supplementary figures and images for: Comparison of Potato Viromes Between Introduced and Indigenous Varieties
Source: Front Microbiol. 2022 May 4;13:809780. doi: 10.3389/fmicb.2022.809780 (PMC9114672; doi:10.3389/fmicb.2022.809780)

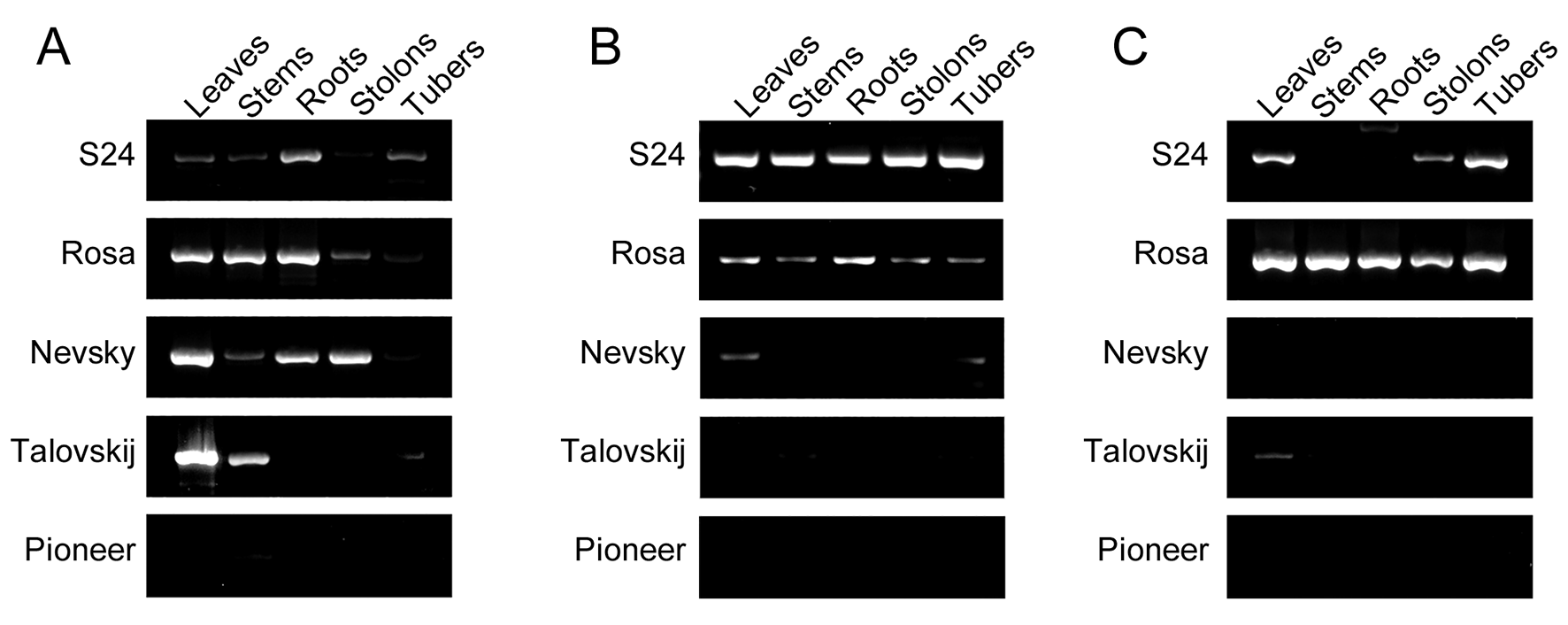

Supplement: Supplementary Figure 1 — Confirmation of 3 identified viruses infecting potato by RT-PCR. Agarose gel electrophoresis results of (A) PVY, (B) PVM, and (C) PVS by RT-PCR with virus-specific primer pairs. Noted: the tubers of “Pioneer”, stolons and roots of “Talovskij” were failed in sampling and further RNA extraction and RT-PCR. [file Image_1.TIF]

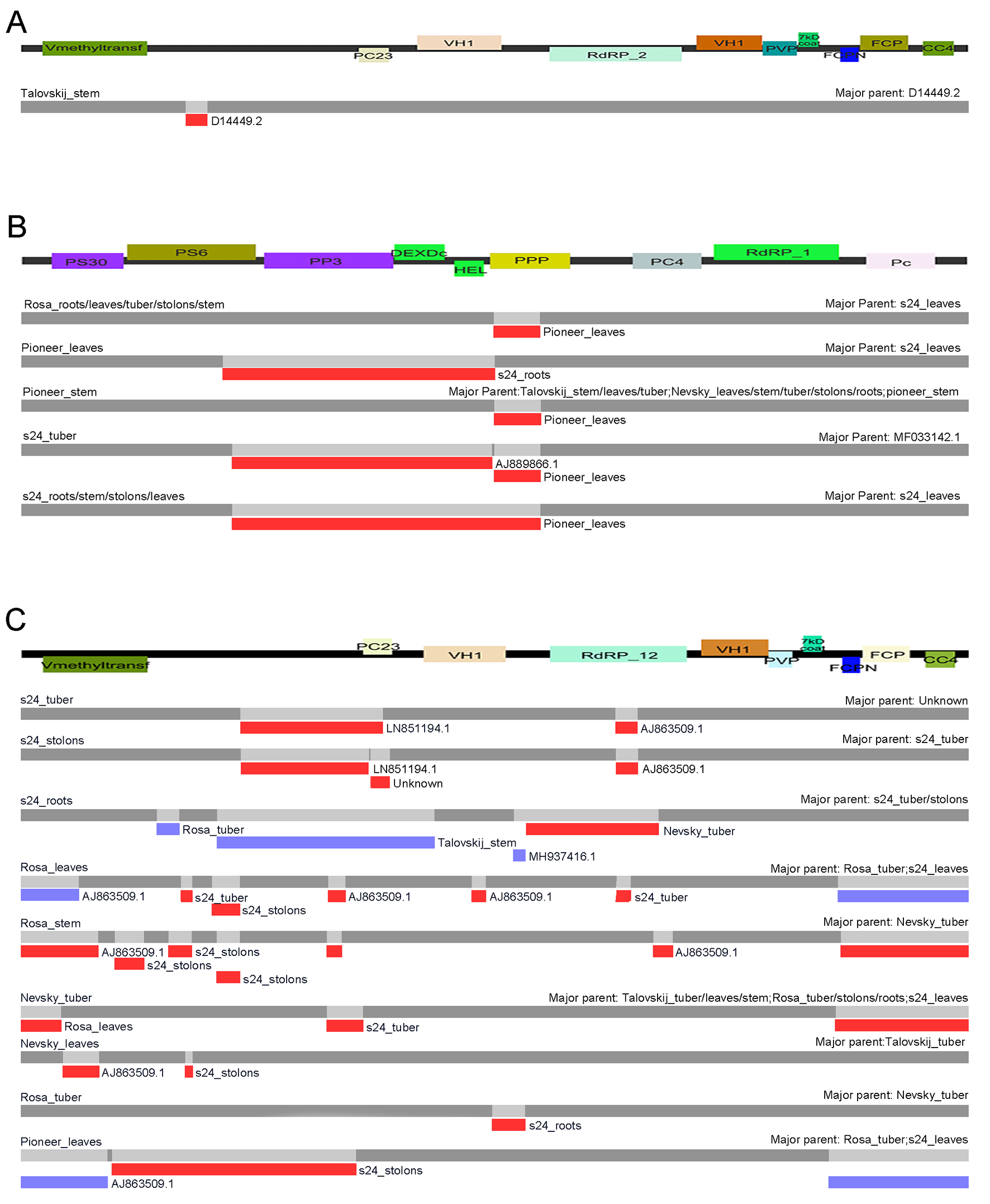

Supplement: Supplementary Figure 2 — A schematic diagram of the recombinant structures in the combined (A) PVM, (B) PVY, and (C) PVS datasets. Regions of recombination are marked by colored bar, and fragments originating from different parents are colored accordingly. [file Image_2.TIF]
